# Mr. Fermor, on Vaccine Inoculation

**Published:** 1801-10

**Authors:** W. Fermor

**Affiliations:** Tusmore


### *To the Editors of the Medical and Phyſical Journal.*

Gentlemen,

In conſequence of ſome occurrences in the Vaccine Inoculation, which have lately taken place in Oxford, I ſhall be much obliged to you to inſert a letter on that ſubject, which I lately received from Dr. Jenner. In proſecuting the effects of a new diſcovery, many circumſtances will naturally occur, which are totally unforeſeen, and which will frequently ſhake the favorable opinions of ſuch as are the greateſt promoters of it. For this reaſon, I particularly wiſhed in a ſmall publication I made laſt year on the ſubject of Vaccine Inoculation, that its practice might be confined to medical men only; and even the moſt enlightened of thefe are fometimes deceived. Too great attention, I am well convinced, cannot be paid to the appearances of the puftule upon the arm, as it indicates whether the diforder has been communicated or not, or whether it be a fpurious one. I mean by fpurious, whether the patient has received the infection of the true Cow-pox; as a wrong fort may be communicated from the matter having been taken at too late a date from a puftule which originally contained the genuine virus, or from the teats of a cow infected with the fpurious fort. The progrefs of the infection will fhew an attentive practitioner, whether he may depend upon the fuccefs of the inoculation. Habit and great attention to the appearance of the characteriftic puftule for fome days after the infertion of the matter, will alone prove how far it has really fucceeded. Some failures therein lately happened in Oxford, the patients having received the Variolous infection after having undergone the Vaccine Inoculation. The fame occurrences have taken place in America, as appears by letters from Dr. Waterhoufe; the particulars of which have come to my knowledge through the polite communications of Dr. Haygarth. During the laft autumn, fimilar circumftances have arifen in Lincolnfhire, as I perceive by the very circumftantial and candid letter from Mr. Fawffett, inferted in your Journal for the month of Auguft laft. It is impoffible for me to conclude my thoughts on this occafion in more appropriate terms than he himfelf has made ufe of. He fays, "The failure of the above cafes, though from the circumftances here mentioned it was, in two of them at leaft, unexpected, ftill cannot be confidered by any one who has paid due attention to the fubject, as invalidating the fecurity of the Cow-Pox Inoculation, where the progrefs of it has been unequivocal. It teaches us, indeed, that a flight deviation from the regular appearances may affect that fecurity; and where a favorable repetition of Inoculation is not in our power, muft of courfe induce us to with-hold our refponfibility for its efficacy in fuch inftances. It ftrongly enforces too the neceffity of minute attention to the progrefs of the local affection, as the only criterion of a fuccefsful reception of it; fever, tendernefs in the axilla, and other more certain fymptoms of conftitutional affection fo frequently being wanting; nor, as appears from one of the above cafes, does a flight appearance of the former afford any additional fecurity, where the local affection is not fatisfactory.

Yours, &c.

